# Conservation of noncoding microsatellites in plants: implication for gene regulation

**DOI:** 10.1186/1471-2164-7-323

**Published:** 2006-12-25

**Authors:** Lida Zhang, Kaijing Zuo, Fei Zhang, Youfang Cao, Jiang Wang, Yidong Zhang, Xiaofen Sun, Kexuan Tang

**Affiliations:** 1 Plant Biotechnology Research Center, Fudan-SJTU-Nottingham Plant Biotechnology R&D Center, School of Agriculture and Biology, Shanghai Jiao Tong University, Shanghai 200030, China; 2 State Key Laboratory of Genetic Engineering, Fudan-SJTU-Nottingham Plant Biotechnology R&D Center, School of Life Sciences, Morgan-Tan International Center for Life Sciences, Fudan University, Shanghai 200433, China

## Abstract

**Background:**

Microsatellites are extremely common in plant genomes, and in particular, they are significantly enriched in the 5' noncoding regions. Although some 5' noncoding microsatellites involved in gene regulation have been described, the general properties of microsatellites as regulatory elements are still unknown. To address the question of microsatellites associated with regulatory elements, we have analyzed the conserved noncoding microsatellite sequences (CNMSs) in the 5' noncoding regions by inter- and intragenomic phylogenetic footprinting in the *Arabidopsis *and *Brassica *genomes.

**Results:**

We identified 247 *Arabidopsis-Brassica *orthologous and 122 *Arabidopsis *paralogous CNMSs, representing 491 CT/GA and CTT/GAA repeats, which accounted for 10.6% of these types located in the 500-bp regions upstream of coding sequences in the *Arabidopsis *genome. Among these identified CNMSs, 18 microsatellites show high conservation in the regulatory regions of both orthologous and paralogous genes, and some of them also appear in the corresponding positions of more distant homologs in *Arabidopsis*, as well as in other plants. A computational scan of CNMSs for known *cis*-regulatory elements showed that light responsive elements were clustered in the region of CT/GA repeats, as well as salicylic acid responsive elements in the (CTT)_n_/(GAA)_n _sequences. Patterns of gene expression revealed that 70–80% of CNMS (CTT)_n_/(GAA)_n _associated genes were regulated by salicylic acid, which was consistent with the prediction of regulatory elements *in silico*.

**Conclusion:**

Our analyses showed that some noncoding microsatellites were conserved in plants and appeared to be ancient. These CNMSs served as regulatory elements involved in light and salicylic acid responses. Our findings might have implications in the common features of the over-represented microsatellites for gene regulation in plant-specific pathways.

## Background

Microsatellites, as one of the major repeat classes, are extremely common in eukaryotic genomes [1]. They are generally thought to result from the mutation effects of replication slippage [2]. Different from the origin of microsatellites from repetitive DNA in animals [3], plant microsatellites show a significant association with nonrepetitive DNA [4]. They can be found abundantly within or near genes in plant genomes, and in particular, some types are significantly enriched within the 5' noncoding regions of plant genes [5-7]. For example, in *Arabidopsis thaliana*, this feature is mostly attributable to the fact that CT/GA and CTT/GAA repeats are more frequently found in 5'-flanks than in other genomic regions, suggesting that they can potentially function as factors in regulating gene expression [7].

For quite a long time, microsatellites were only considered as genetic markers in DNA fingerprinting and diversity studies due to the extensive length polymorphisms. However, recent findings show that some of them act as *cis*-regulatory elements which can be recognized by transcription factors [8,9]. It has been well known for so-called GAGA elements, comprising the dinucleotide repeat sequence (GA)_n _to be present in promoters regulating numerous developmental genes in animals [10,11]. Similarly, the (GA)_n _sequences in regulatory regions of some plant genes can also be recognized by GAGA-binding factors [12-14], and more generally, the GA-rich element, a more complex 9 base pairs (bp) based (GA)_n _repeat, has been shown to have protein-binding affinity [15]. Another major microsatellite in plants, the trinucleotide repeat sequence (GAA)_n _presented within 5'UTR of *ntp303 *was found important in the modulation of transcription and translation efficiency [16]. Furthermore, some unusual phenotypic variations were found to be associated with the length of 5' noncoding microsatellites. A typical example was reported by Bao and his colleagues that variation in the number of CT/GA repeats in the 5'UTR of the *waxy *gene was correlated with amylose content in rice [17]. Although the mechanism is still unclear, the microsatellite length polymorphism is thought to affect the expression of the related genes of amylose synthesis.

Regions of DNA involved in gene regulation are expected to exhibit sequence conservation between related species over evolutionary time due to functional constraints. It has been recognized that comparative analyses of noncoding DNA sequences in multiple species, known as phylogenetic footprinting, can help identify conserved putative regulatory elements [18]. Successful identification of conserved noncoding sequences in comparisons among different grass genomes and cruciferous species, as well as between closely related genomic sequences from *Arabidopsis *and *Brassica *species has provided some good references for discovery of Conserved Noncoding Microsatellite Sequences (CNMSs) by phylogenetic footprinting in plants [19-22].

If microsatellites are important for regulating gene expression, they should be conserved in the homologous promoters through gene duplication or speciation during plant evolution. To address the question of microsatellites associated with gene regulatory elements, we used inter- and intragenomic phylogenetic footprinting to analyze the dominant microsatellites in the 5' noncoding regions of *Arabidopsis *and *Brassica oleracea *genes for CNMSs. About 10% of 5' noncoding CT/GA and CTT/GAA repeats are conserved in the *Arabidopsis *genome, and they are preferentially involved in gene regulation in plant-specific pathways.

## Results

### Distribution of microsatellites in different genomic regions

**Figure 1:**
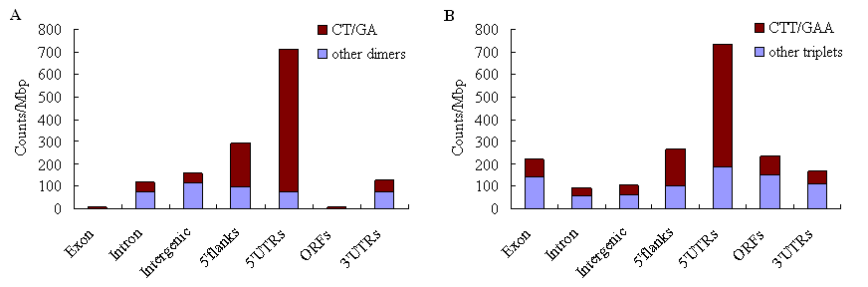
**Distribution of microsatellites in different genomic regions in *Arabidopsis***. (A) Frequencies of dinucleotide repeats in different genomic fractions. Other dimers include all dimers except CT/GA repeats. (B) Frequencies of trinucleotide repeats in different genomic fractions. Other triplets include all triplets except CTT/GAA repeats. 5'-flanks correspond to the 500 bp sequences upstream of the initiation codon.

The characteristics of microsatellite occurrences were surveyed among the different genomic regions in the *Arabidopsis *genome. It was obvious that microsatellites were found to be highly abundant in the regulatory regions, and the over-representation of CT/GA and CTT/GAA repeats contributed most to the increase of microsatellites in these regions (Figure 1A, 1B). This preference of CT/GA and CTT/GAA repeat occurrences indicated that they might have the role in regulating genes.

### Conservation of microsatellites in *Arabidopsis*

**Figure 2:**
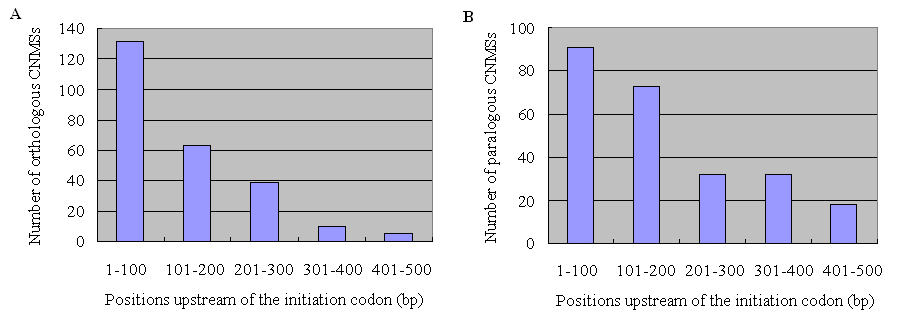
**Distribution of (A) orthologous and (B) paralogous CNMSs in the 5' noncoding regions *in Arabidopsis***. The position is indicated as segments of 100 bp.

Regulatory sequence elements within promoter DNA are often short, orientation independent and contain frequent gaps of variable size. Thus, we determined the conserved noncoding microsatellite sequences as candidate regulatory elements based upon the following criteria: that there were at least 6-bp overlapping regions of the corresponding microsatellites between the aligned sequences. According to the criteria, we identified 247 *Arabidopsis-Brassica *orthologous CNMSs and 122 *Arabidopsis *paralogous CNMSs [see Additional file 1], involving 491 CT/GA and CTT/GAA repeats respectively (Table 1), which accounted for 10.6% of these types located in the 500-bp regions upstream of coding sequences in the *Arabidopsis *genome. These CNMSs do not randomly occur in different noncoding regions and they tend to be found more frequently near the initiation codon (Figure 2A, 2B).

**Figure 3:**
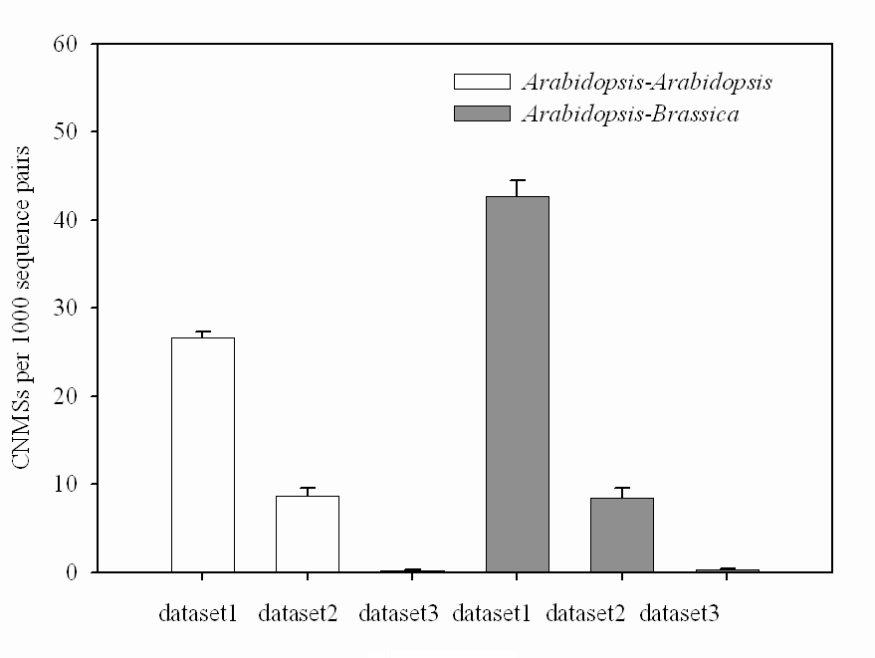
**Occurrences of CNMSs in the random datasets**. Dataset 1, dataset 2 and dataset 3 respectively correspond to the 1000 homologous pairs, the 1000 shuffled pairs of noncoding sequences and the 1000 random pairs of genomic sequences in the analysis. Sequence length is 500 bp. Occurrences of CNMSs were analyzed in analogous manner for 10 different random sets with equal data size. Means of CNMS occurrences are indicated on the y axis, and error bars represent SEs.

In order to validate the above study and to ensure that the observation of CNMSs was not simply due to its over-representation in plant genomes, a similar analysis was carried out on three different random datasets, i.e. the 1000 homologous pairs of 5' noncoding sequences in *Arabidopsis *as dataset 1, the 1000 randomly shuffled pairs of 5' noncoding sequences as dataset 2 and the 1000 random pairs of genomic DNA sequences as dataset 3, as well as the three corresponding datasets of *Arabidopsis *and *Brassica *sequence pairs with the same data size. Figure 3 showed the frequencies of CNMS (CT/GA)_n _and (CTT/GAA)_n _in dataset 1, dataset 2 and dataset 3, respectively. Obviously, there was very little probability that CNMSs were found in the 500-bp genomic DNA sequence pair by chance. In contrast with the random pairs of noncoding sequences, the homologous noncoding sequences showed significant high in the frequency of CNMS occurrences. Taken together, these tests indicated that some microsatellites in regulatory regions were conserved from common ancestors during plant evolution.

### Evolution of conserved microsatellites in *Arabidopsis*

**Figure 4:**
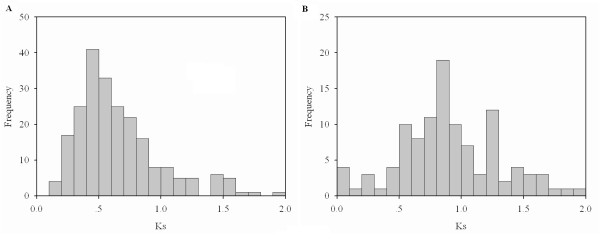
**Distribution of synonymous substitution rate (*Ks*) for CNMS gene sets**. (A) Distribution of *Ks *values for *Arabidopsis-Brassica *orthologous CNMS gene set. A clear peak for *Ks *values of 0.4 to 0.5. (B) Distribution of *Ks *values for paralogous CNMS gene set in *Arabidopsis*. Two clear peak for the *Ks *values of 0.8 to 0.9 and 1.2 to 1.3.

To gain insight into the evolutionary relationship of *Arabidopsis-Brassica *and *Arabidopsis-Arabidopsis *CNMSs, the synonymous substitution rate (*Ks*) was calculated for the corresponding gene pairs. For *Arabidopsis-Brassica *orthologous CNMS gene pairs, the frequency distribution showed a clear peak for *Ks *values of 0.4 to 0.5 (Figure 4A), suggesting that these CNMSs were conserved from a common ancestor over a 15 million years (Myr) period, which was consistent with the divergence time frame estimated at 14.5 to 20.4 Myr based on mitochondrial DNA data [23]. On the other hand, we noticed two peaks in the *Ks *distribution of *Arabidopsis *paralogous CNMS gene pairs, and the *Ks *values were 0.8 to 0.9 and 1.2 to 1.3, respectively (Figure 4B). The former group contained most of the paralogous CNMSs which were originated from large scale gene duplication over 28 Myr ago, which was consistent with the recent polyploidization event during evolution of the *Arabidopsis *genome [24]. The latter group were duplicated from the common ancestor over 42 Myr ago, which probably occurred at the time of the divergence of brassicaceae family [25].

**Figure 5:**
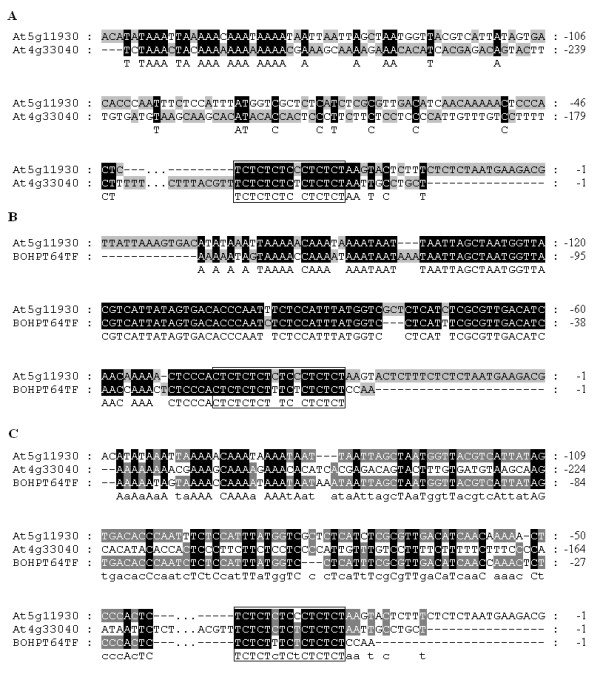
**An example of microsatellites conserved among paralogous and orthologous genes**. Sequence alignments and positions for the microsatellites conserved among (A) *Arabidopsis *paralogous genes, (B) *Arabidopsis-Brassica *orthologous genes and (C) homologous genes from *Arabidopsis *and *Brassica*. Boxed regions indicate CNMSs. Dots indicate omits of alignment. Nucleotide positions are given relative to the initiation codon.

The results from the evolutionary relationships of *Arabidopsis-Brassica *and *Arabidopsis-Arabidopsis *CNMSs suggested that most paralogous CNMSs pre-dated the divergence of the two species; hence, many paralogous CNMSs in *Arabidopsis *were likely to find their counterparts in *Brassica*. Further comparisons of paralogous and orthologous genes from *Arabidopsis *and *Brassica *were made for common CNMSs (Figure 5A, 5B). With the same criteria, we identified 18 CNMSs found in *Arabidopsis *paralogous pairs that also were coincident with CNMSs from at least one orthologs in *Brassica *(Table 2). We called these conserved elements, shared among paralogous and orthologous genes, Ultra-CNMSs. An example of such Ultra-CNMSs was shown in Figure 5C, and the three homologous CT repeats were highly conserved from a common ancestor over 48 Myr.

### Conservation of microsatellites in plants

**Figure 6:**
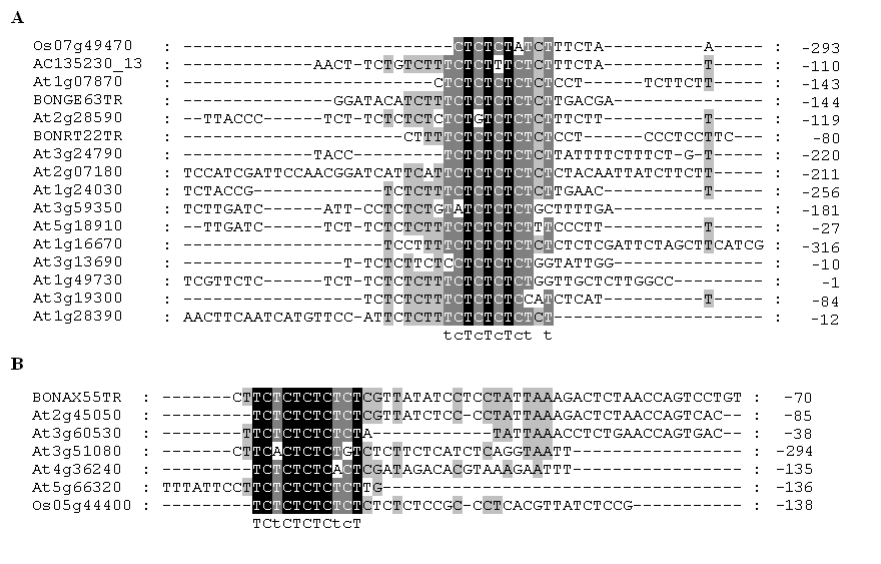
**Conservation of microsatellites in plants**. (A) Sequence alignments for the CNMS conserved among the homologous protein kinase genes from *Arabidopsis, Brassica, Medicago *and rice. Atlg07870, At2g28590, BONGE63TR, BONRT22TR, AC135230_13 and Os07g49470 are orthologs for the four species. Sequences of *Medicago *(AC135230_13) and rice (Os07g49470) obtained from TIGR plant genome sequence database. (B) Sequence alignments for the CNMS conserved among the homologous GATA transcription factor genes from *Arabidopsis, Brassica *and rice. BONAX55TR and At2g45050 are orthologs for *Brassica *and *Arabidopsis*. The homologous gene Os05g44400 from rice.

As expected, analysis of regulatory regions of related gene families revealed that many Ultra-CNMSs were conserved across a number of more distantly homologous genes in Brassicaceae species and other plants. Figure 6A showed that CNMSs (CT)_n _were conserved among orthologous genes from *Arabidopsis, Brassica, Medicago *and rice, as well as among more distantly paralogous genes in *Arabidopsis*. These genes are representatives of a larger family of transmembrane receptor kinases and related non-transmembrane kinases in plant genomes. Many of them arised from a common ancestor of dicots and monocots. Another striking CNMS was found in the regulatory regions of GATA transcription factor genes from *Brassica, Arabidopsis *and rice. Of 14 members in subfamily I in the *Arabidopsis *genome [26], five of them have the same CNMSs found in their regulatory regions (Figure 6B). These CNMS associated transcription factor genes that fell into two subgroups indicated they diverged before the dicotyledonous and monocotyledonous plants [26]. It was obvious that these Ultra-CNMSs had been passed down from a common ancestor of dicots and monocots under extreme purifying selection for more than 170 Myr [27].

### Annotation enrichment and depletion of CNMS associated genes

**Figure 7:**
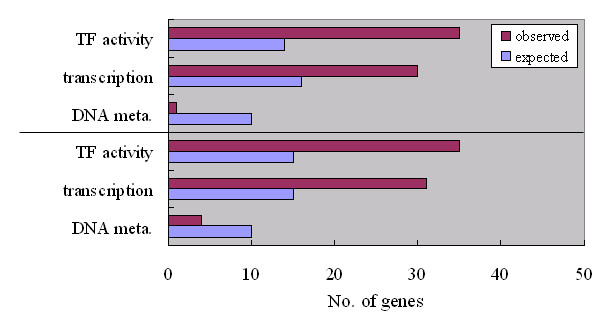
**Annotation enrichment and depletion of CNMS associated genes**. In the top half of the figure, the maroon bars ("observed") give the numbers of orthologous CNMS genes that are annotated in the *Arabidopsis *GO database with molecular function "transcription factor activity" or biological process "transcription regulation" and "DNA metabolism". The blue bars ("expected") give the number of genes that one would expect to obtain if the same number of genes were chosen at random among all genes annotated in the relevant database. The bottom half of the figure gives similar information for paralogous CNMS genes in *Arabidopsis*.

We tested whether CNMSs in genes were influenced by the function of the proteins they encode. There were 206 *Arabidopsis-Brassica *and 194 *Arabidopsis-Arabidopsis *CNMS associated genes with known function in the *Arabidopsis *genome. We looked for categories of biological process and molecular function defined in the Gene Ontology (GO) database that were significantly enriched or depleted in these genes [28,29]. These CNMS associated genes showed significant functional enrichment for transcription factor activity (P < 1.8 × 10^-7 ^for orthologous CNMS genes, and P < 7.7 × 10^-7 ^for paralogous CNMS genes, against all GO annotated *Arabidopsis *genes) and transcription (P < 4.5 × 10^-4 ^for orthologs and P < 6.6 × 10^-5 ^for paralogs) (Figure 7), and genes that performed the functions of the two types accounted for about 23% of all known genes. However, they were obviously depleted for DNA metabolism (P < 2.2 × 10^-4 ^for orthologous CNMS genes, and P < 1.8 × 10^-2 ^for paralogous CNMS genes). These findings suggested that CNMSs might be specifically associated with regulation of transcription at the DNA level, but not involved in DNA metabolism.

### CNMSs as regulatory elements in plants

To further investigate the regulatory nature of these CNMSs, we employed a computational method to discover *cis*-elements that were similar to function assigned elements based on the PlantCARE [30,31] and PLACE databases [32,33]. The identification of *cis*-elements showed that some binding sites were clustered in the consevered microsatellite regions, and these regulatory elements were involved in plant-specific functions in response to some environmental stimuli (Table 3). The CNMS (CT)_n _include the TCTCtCT sequences similar to the TCCC motif known as part of conserved DNA module array AtpCD-CMA involved in light responsiveness [34,35]. Another function of CNMS (CT)_n _may be as an enhancer due to the same motif (TCTCTCTCT) found in a 60-nt region downstream of the transcription start site of the CaMV 35S RNA, which can enhance gene translation in plant protoplasts [36]. As complementary sequences to (CT)_n_, (GA)_n _serve as regulatory element having similar functions, which contain a series of overlapped GAG motifs (AGAGAGa) involved in light regulation [35,37]. In soybean, it is clear that the 18-bp GAGA element sequence within the *Gsal *promoter can be recognized by GBP encoded by a light-regulated gene [12]. The CNMS (CTT)_n _contain sequences similar to the TCA-element (TCATCTTCTT) which is a binding site for salicylic acid-inducible proteins [38]. Similarly, the CNMS (GAA)_n _contain AGAA sequences having the characteristic of the core recognition sequence (tcAGAAgagg) for salicylic acid-responsive genes [39].

**Figure 8:**
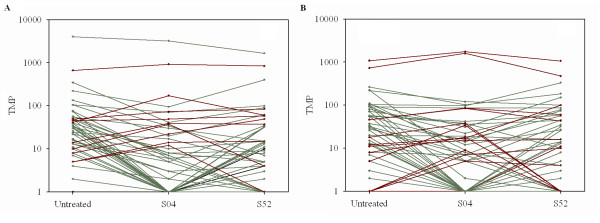
**Expression patterns of (A) orthologous and (B) paralogous CNMS (CTT)_n_/(GAA)_n _associated genes in *Arabidopsis *leaves with salicylic acid treatment**. Genes were inhibited after 4 hours of treatment with salicylic acid (green), induced after 4 hours of treatment with salicylic acid (red) and kept inactive until 52 hours of treatment with salicylic acid (black). The gene expression levels were estimated by *Arabidopsis *MPSS data from three different libraries generated by untreated leaves and treated leaves 4 (S04) and 52 hours (S52) after salicylic acid treatment, respectively. TPM is normalized value in transcripts per million for each signature in the library.

**Figure 9:**
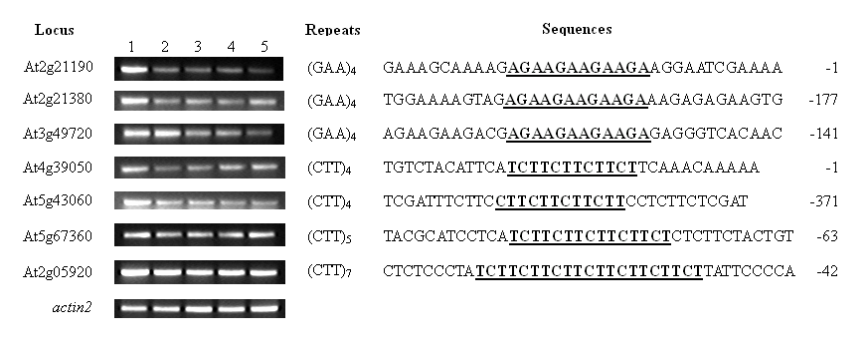
**Expression patterns of *Arabidopsis *CNMS (CTT)_n_/(GAA)_n _associated genes and their related sequence information**. The expression of genes were assayed by RT-PCR. Lanes 1–5: control (untreated), 1, 4, 12 and 48 hours after treatment with 1 mM salicylic acid, respectively. The *actin2 *gene was used as an internal control in the RT-PCR reaction.

Although CNMSs (CT)_n_/(GA)_n _and (CTT)_n_/(GAA)_n _are similar to known regulatory elements, most of them have no experimental verification for their functions. Therefore, all CNMS (CTT)_n_/(GAA)_n _associated genes were selected to investigate their changes in expression levels after the treatment of salicylic acid. The abundance of gene transcripts evaluated by the MPSS showed these CNMS (CTT)_n_/(GAA)_n _associated genes had distinct expression characters with salicylic acid treatment (Figure 8A, 8B). About 70–80% of CNMS (CTT)_n_/(GAA)_n _associated genes in *Arabidopsis *leaves were regulated by salicylic acid, while others were undetectable with and without salicylic acid treatment. Among these salicylic acid-responsive genes, only about 15–23% of them were up-regulated by salicylic acid, and most of them were inhibited after the treatment. Seven CNMS (CTT)_n_/(GAA)_n _associated genes were additionally analyzed for expression patterns after salicylic acid treatment. The RT-PCR showed that these investigated genes, excepted At2g05920 and At5g67360, were obviously down-regulated by salicylic acid (Figure 9), which were consistent with the patterns of gene expression from the *Arabidopsis *MPSS database. According to the expression patterns by RT-PCR, we found the preliminary correlation between repeat number of CTT/GAA motif and gene in response to salicylic acid. The (CTT)_4_/(GAA)_4 _sequences were associated with gene down-regulation with salicylic acid stimulus, but the (CTT)_5 _and (CTT)_7 _associated genes were not obviously regulated by salicylic acid. These findings implied that regulation of CNMS associated gene expression by salicylic acid might be dependent on the number of CTT/GAA repeats.

## Discussion

Microsatellites (CT)_n_/(GA)_n _and (CTT)_n_/(GAA)_n _are well presented in the *Arabidopsis *genome, and in particular, they are preferentially located within the 5' noncoding regions. In this study, we identified 491 conserved CT/GA and CTT/GAA repeats for candidate regulatory elements by inter- and intragenomic phylogenetic footprinting. These CNMSs tend to occur within these regions near the initiation codon with the preference of CT and CTT motifs, which are consistent with the characteristic of pyrimidine-rich repeat distribution in these regions [5,7]. Another striking feature of CNMS distribution is that they are rarely found in the peri-centromeric regions; in contrast, their related genes are always clustered in chromosome arms (data not shown). The reasons for the absence of CNMS on peri-centromeric regions are still unclear, but CNMS associated genes occurring in clusters on chromosome arms is probably attributable to co-expression.

Microsatellites generally evolve rapidly, but there are about 10% of 5' noncoding CT/GA and CTT/GAA repeats which show high conservation in occurrences and appear to be ancient. In particular, the Ultra-CNMSs have been under purifying selection for more than 42 Myr, and some of them for at least 170 Myr. This conservation may be explained by function constraint so that many homologous genes have the corresponding microsatellite sequences in their regulatory regions. Most microsatellites of CT/GA and CTT/GAA types seem to be originated by recent mutations under positive selection [4,7], which lead to the significant over-representation of microsatellites in the 5' noncoding regions compared with other genomic fractions. The reasons of positive selection for some repeat occurrences are still unknown. However, at least, they may provide opportunities for rapid adaptive changes in these regulatory regions or play specific roles in gene regulation.

It is well known that intergenomic phylogenetic footprinting is an effective method for the discovery of regulatory elements in a set of orthologous noncoding regions from multiple species [18-22]. In plant genomes, intragenomic phylogenetic footprinting represents another powerful strategy to detect regulatory elements due to the facts that most plant genomes are rich in duplicated genes and large fractions of these gene pairs share transcriptional characteristics [40]. Although detection of the full complement of *cis*-elements is not feasible by this approach due to potential acquisition and loss of individual regulatory elements between duplicated promoters, we can readily identify several specific regulatory elements which show high conservation in duplicated genes. Using this approach, we have successfully identified 122 *Arabidopsis *CNMSs as candidate regulatory elements of plant-specific function. Most of paralogous CNMSs were originated from the recent polyploidization event before the divergence between *Arabidopsis *and *Brassica *[23], implying that they might be conserved with their counterparts in *Brassica*. We compared the data generated by inter- and intragenomic phylogenetic footprinting and found 18 CNMSs highly conserved in both orthologous and paralogous sequences. The number of the identified ultra-CNMSs may be underestimated for the incomplete reference genome sequences of *Brassica *or the false orthologous relationships. These conserved microsatellites occurring among three or more homologous genes provides greater evidence that these CNMS are likely to be significant in gene regulation.

Functional annotation showed that CNMS associated genes were obviously depleted for DNA metabolism, such as DNA replication, DNA recombination and DNA repair. It is possible that genes that are essential for survival, lack CNMSs within their 5' noncoding regions because these genes do not need some specific regulatory elements. In contrast, these CNMS genes are preferentially associated with regulation of transcription in plants. CNMSs serve as regulatory elements and their related genes can be responsive to one or more forms of environmental stimuli (Table 3). The functional biases imply that CNMS associated genes (e.g. transmembrane receptor kinase genes and transcription factor genes) encoding proteins are involved in upstream pathways of defense responses in plants.

Although GAGA elements are known to be involved in the regulation of numerous developmental genes in animals [10,11], we believe that CNMSs (CT)_n_/(GA)_n _are likely to be associated with transcriptional regulation in light signaling pathways in plants [35]. These CNMSs are often found in a number of different light-regulated genes [12,41]. Although expression of most CNMS (CT)_n_/(GA)_n _associated genes was not significantly changed with light/dark transitions, three Ultra-CNMSs related genes (At5g52430, Atlg21920 and At3g62650) were obviously induced with longer periods of darkness according to microarray gene expression data of 7800 unique *Arabidopsis *genes [42]. This was consistent with the fact that about 9% of these CNMS genes were significantly down-regulated, while only 2% of them were up-regulated for light by a whole-genome expression analysis in seedling of *Arabidopsis *[43]. It is possible that the expression level changes of most CNMS (CT)_n_/(GA)_n _associated genes are not obvious under light since they are always in upstream of related pathways. However, CNMSs (CT)_n_/(GA)_n_, at least parts of them, may be the binding sites for *trans*-acting regulators involved in light signaling pathways and their associated genes can be induced under darkness.

Salicylic acid is well known as an important signaling molecule involved in both locally and systemically induced disease resistance responses [44]. Many salicylic acid responsive genes have been found in plant defense pathways. The CNMS (CTT)_n_/(GAA)_n _associated genes exhibit distinct expression characters with salicylic acid treatment, implying that they may be associated with a range of different stresses [38]. CNMSs (CTT)_n_/(GAA)_n _as regulatory elements regulating gene expression are associated with the repeat number in salicylic acid signaling pathways. They may not act as isolated transcription factor binding sites to regulate gene expression. Instead, they are likely to co-operate with other elements to perform complex regulatory functions in transcription. Perhaps some of them may perform roles in RNA interference by forming RNA duplexes with complementary antisense microsatellite sequences, which lead to quite a few CNMS genes whose transcripts are undetectable in *Arabidopsis *leaves.

## Conclusion

Microsatellites (CT)_n_/(GA)_n _and (CTT)_n_/(GAA)_n _are preferentially associated the 5' noncoding regions in the *Arabidopsis *genome. Parts of them are conserved among the homologous genes and appear to be ancient. The computational prediction and gene expression analysis indicated that CNMSs (CT)_n_/(GA)_n _and (CTT)_n_/(GAA)_n _acted as regulatory elements involved in light and salicylic acid responses. From our analysis, the presence of CT/GA and CTT/GAA repeats in regulatory regions may be particularly useful as a guide for further experiments of plant regulatory networks in response to environmental stimulus.

## Methods

### Plant materials

The *Arabidopsis *plants were grown in soil in a growth chamber at 20°C with 8 hours of light for 40 days. Plants were sprayed to run-off with 1 mM salicylic acid in 0.5% dimethyl sulfoxide (DMSO) for different time scales. One, four, twelve and forty-eight hours post-treatment, leaves were cut and harvested respectively, quick-frozen in liquid nitrogen, then stored at -80°C. Total RNA was later extracted using Plant RNA Mini Kit (Watson Biotechnologies INC., China).

### Sequence data sources

The annotated sequences of the five chromosomes of *Arabidopsis *(accession numbers: NC_003070, NC_003071, NC_003074, NC_003075, and NC_003076, updated 25-JAN-2005) were downloaded from the Genomes Division of GenBank [45,46]. Intergenic regions were defined as being a part of DNA from the end of the last exon of one gene to the beginning of the first exon of the following gene. A set of 16223 full-length cDNA sequences containing both 5' and 3'UTRs for *Arabidopsis *was extracted from the TAIR database [47]. The preliminary sequences of *Brassica *genome were obtained from The Institute for Genomic Research website [48].

### Identification of orthologous and paralogous gene pairs

To identify putative *Arabidopsis-Brassica *orthologous gene sets, each preliminary sequence from *Brassica *was searched against 1-kb sequences (fragments from the position -500 to +500 relative to the translation initiation) of all genes from *Arabidopsis *using BLASTN [49] and then the fragments from *Brassica *were clustered according to the best match gene of the *Arabidopsis *genome. Conversely, each 1-kb gene sequence from *Arabidopsis *was searched against the contigs from *Brassica*. Two sequences were defined as orthologs if each of them was the best hit of the other in the aligned regions and if the expect value (E) was <le-10. A list of the identified *Arabidopsis-Brassica *orthologs in the study is provided as supplementary data [see Additional file 2].

For identifying the paralogous gene pairs from a recently common ancestor in the *Arabidopsis *genome, each annotated coding sequence was searched against all other coding sequences using BLASTN. The best pair was considered significant if each of them was the best hit of the other and the expect value was <le-10. A file of the list of the paralogous gene pairs is included as supplementary data [see Additional file 3]. To avoid the negative conservation of microsatellites caused by the effects of insufficient randomizing mutations, the tandemly repeated gene pairs separated by less than 25 intermediate genes were ignored in further analysis.

### Microsatellite detection

Microsatellites were found in sequences using the modified Sputnik repeat-finder [50]. Di-and trinucleotide repeats were identified when a total size of at least 12-bp, allowing up to about 10% deviation from a perfect repeat. Repeat motifs consisting of different frames (e.g. GAA, AGA and AAG) were regarded as the same type of repeat.

### Identification of CNMSs

Because gene fragments of *Brassica *were derived from preliminary contigs with no annotated open reading frames, each pair of *Arabidopsis-Brassica *sequences were aligned using DiAlign2 with translation option to identify the 5' noncoding sequences and coding regions in the *Brassica *orthologs [51]. The 5' noncoding sequence pairs were aligned using DiAlign2 for finding conserved microsatellites. To exclude nonspecific alignments, a stringent threshold parameter of 3 was used. The CNMSs were identified when the corresponding loci had at least 6-bp overlapping sequences between the aligned microsatellite sequences.

### Selection of random data sets

To ensure that CNMSs were not to occur by chance, we used two different datasets of random pairs as negative controls to validate the results. One control dataset contained 1000 random pairs of 500-bp upstream noncoding sequences in the *Arabidopsis *genome, and another control dataset of 1000 pairs was randomly generated from the 500-bp sequences of *Arabidopsis *genomic DNA fragments. The reference dataset of equal data size was randomly selected from the 500-bp paralogous noncoding sequence pairs in *Arabidopsis*. The 1000 paralogous pairs, the 1000 shuffled pairs of noncoding sequences and the 1000 random pairs of genomic sequences were respectively referred as dataset 1, dataset 2 and dataset 3 in further analysis. Similarly, three corresponding datasets of *Arabidopsis *and *Brassica *sequence pairs were generated with the same data size. The dataset 1 consisted of 1000 *Arabidopsis-Brassica *orthologous pairs of 5' noncoding sequences, and the dataset 2 contained 1000 random pairs of *Arabidopsis *and *Brassica *upstream noncoding sequences, and the dataset 3 of 1000 pairs was randomly generated from the *Arabidopsis *and *Brassica *genomic DNA sequences. The same criteria of CNMS detection was applied in the test. Occurrences of CNMSs were analyzed in analogous manner for 10 different random sets with equal data size.

### Estimation of duplication and speciation time

We used the level of synonymous substitution of CNMS associated coding sequences to estimate the *Ks *of CNMSs. For each pair of CNMS associated genes, the two protein sequences were aligned by ClustalW, and the resulting alignment was then used as a guide to align the nucleotide sequences [52]. After removing gaps, the level of synonymous substitution was estimated using the yn00 program in PAML [23]. The time of divergence (T), between two sequences was calculated from this as T = *Ks*/2λ, where *Ks *is the fraction of synonymous substitutions per synonymous site and λ is the mean rate of synonymous substitution. The estimate value for λ in dicots is 1.5 synonymous substitutions per 10^8 ^years [53].

### Estimation of gene expression level

Gene expression level was estimated using the data from the Massively Parallel Signature Sequencing (MPSS) database of *Arabidopsis *[54,55]. The MPSS data of three different libraries was generated from untreated leaves and treated leaves 4 and 52 hours after salicylic acid treatment, respectively. For the three libraries, a total of 9,081,200 17-bp signatures were obtained in multiple sequencing runs and in two sequencing frames. The abundance for each signature was normalized to transcripts per million (TPM) to facilitate comparisons across libraries.

RT-PCR of *Arabidopsis *CNMS associated genes was conducted using the one-step RNA PCR kit (TaKaRa) with gene specific primers [see Additional file 4]. The 0.5 μg total RNA was used as the template to be amplified with the following program: an initial 50°C for 30 min and 94°C for 2 min, followed by 25 cycles of 94°C for 30s, 54°C for 30s and 72°C for 1 min. The house-keeping gene *actin*2 (At3gl8780) was used as an internal control in RT-PCR reaction.

## Authors' contributions

ZL designed and conducted the study on microsatellite detection, identification of CNMSs, comparative genome analysis, evolution, and drafted the manuscript. ZK and ZF provided the plant materials and participated in gene expression analysis. CY performed database searches. WJ and ZY participated in data analysis and manuscript revision. SX and TK participated in research design and in the drafting of the manuscript. All authors read and approved the final manuscript

## Supplementary Material

Additional file 1 The full list of pairs of conserved noncoding microsatellites identified in this study. Click here for file

Additional file 2 List of the orthologs between *Arabidopsis *and *Brassica*. Click here for file

Additional file 3 List of the paralogous gene pairs in the *Arabidopsis *genome. Click here for file

Additional file 4 The primer sequences used for RT-PCR. Click here for file

## Figures and Tables

**Table 1:** Summary of *Arabidopsis-Brassica *and *Arabidopsis-Arabidopsis *CNMSs

	CNMSs				Total	*Arabidopsis *CNMS genes
			
	(GA)_n_	(CT)_n_	(GAA)_n_	(CTT)_n_		
*Arabidopsis-Brassica*	51	131	30	35	247	242
*Arabidopsis-Arabidopsis*	19	59	10	34	122	234

**Table 2:** Ultra-CNMSs in *Arabidopsis-Brassica *orthologs and *Arabidopsis *paralogs

CNMSs	Orthologs		Orthologs		Function description
			
	*Arabidopsis*	*Brassica*	*Arabidopsis*	*Brassica*	
(GA)_n_	At2g16780	BOMMT72TR	At4g35050		WD-40 repeat protein
(GA)_n_	At4g25620	gn1ltil104045340	At5g52430		hydroxyproline-rich glycoprotein protein
(GAA)_n_	Atlg12420	BONGX39TF	At4g22780		ACT domain protein
(GAA)_n_	At4g29000	BOHVH87TR	At2g20110		TSO1-like CXC domain protein
(CT)_n_	Atlg07870	BONGE63TR	At2g28590	BONRT22TR	protein kinase
(CT)_n_	Atlg14870	BONHS20TF	At5g35525		unknown
(CT)_n_	Atlg68360	BOIHI85TR	Atlg67030		zinc finger
(CT)_n_	Atlg77660	BOOAX17TF	Atlg21920		phosphatidylinositol-4-phos phate 5-kinase-related
(CT)_n_	At2g28890	BOHQE28TR	Atlg07630		protein phosphatase 2C
(CT)_n_	At2g45050	BONAX55TR	At3g60530		zinc finger
(CT)_n_	At2g47440	BOHWG09TF	At3g62570		heat shock protein
(CT)_n_	At3g06760	BOICE92TR	At5g49230		drought-responsive protein
(CT)_n_	At2g47485	BOIFI37TR	At3g62650		unknown
(CT)_n_	At3g22270	BOGAX66TF	At4g14990		unknown
(CT)_n_	At4g34720	BOHJQ57TF	At2g16510		vacuolar ATP synthase
(CT)_n_	At5g01240	BOHGK06TF	At2g38120		amino acid permease
(CT)_n_	At5g11930	BOHPT64TF	At4g33040		glutaredoxin protein
(CTT)_n_	At5g27430	gn1ltil103985703	At3g05230		signal peptidase subunit

**Table 3:** Prediction of CNMSs serve as regulatory elements *in silico*

CNMSs	Method	Motif Name	Recognized Sequence	Motif Function
(GA)_n_	PlantCARE	GAG-motif	AGAGAGa	part of a light responsive element [35,37]
	PLACE	GAGAGMGSA1	(GA)_9_	Binding site for GAGA-binding factor, and *Gbp *is a light-responsive gene [12].
(GAA)_n_	PlantCARE	TCA-element	aAGAAgaaga	salicylic acid responsive element [39]
(CT)_n_	PlantCARE	TCCC-motif	TCTCtCT	part of a light responsive element [34,35].
	PLACE	CTRMCAMV35S	TCTCTCTCT	CT-rich motif found in a 60-nt region downstream of the transcription start site of the CaMV 35S RNA; Can enhance gene expression [36].
(CTT)_n_		TCA-element	TCtTCTTCTT	salicylic acid responsive element [38]

## References

[B1] Toth G, Gaspari Z, Jurka J (2000). Microsatellites in different eukaryotic genomes: survey and analysis. Genome Res.

[B2] Levinson G, Gutman GA (1987). Slipped-strand mispairing: a major mechanism for DNA sequence evolution. Mol Biol Evol.

[B3] Nadir E, Margalit H, Gallily T, Ben-Sasson SA (1996). Microsatellite spreading in the human genome: evolutionary mechanisms and structural implications. Proc Natl Acad Sci USA.

[B4] Morgante M, Hanafey M, Powell W (2002). Microsatellites are preferentially associated with nonrepetitive DNA in plant genomes. Nat Genet.

[B5] Fujimori S, Washio T, Higo K, Ohtomo Y, Murakami K, Matsubara K, Kawai J, Carninci P, Hayashizaki Y, Kikuchi S, Tomita M (2003). A novel feature of microsatellites in plants: a distribution gradient along the direction of transcription. FEBS Lett.

[B6] Lawson MJ, Zhang L (2006). Distinct patterns of SSR distribution in the Arabidopsis thaliana and rice genomes. Genome Biol.

[B7] Zhang LD, Yuan DJ, Yu SW, Li ZG, Cao YF, Miao ZQ, Qian HM, Tang KX (2004). Preference of simple sequence repeats in coding and non-coding regions of *Arabidopsis thaliana*. Bioinformatics.

[B8] Iglesias AR, Kindlund E, Tammi M, Wadelius C (2004). Some microsatellites may act as novel polymorphic *cis*-regulatory elements through transcription factor binding. Gene.

[B9] Martin P, Makepeace K, Hill SA, Hood DW, Moxon ER (2004). Microsatellite instability regulates transcription factor binding and gene expression. Proc Natl Acad Sci USA.

[B10] Bevilacqua A, Fiorenza MT, Mangia F (2000). A developmentally regulated GAGA box-binding factor and Sp1 are required for transcription of the hsp70.1 gene at the onset of mouse zygotic genome activation. Development.

[B11] Busturia A, Lloyd A, Bejarano F, Zavortink M, Xin H, Sakonju S (2001). The MCP silencer of the Drosophila Abd-B gene requires both pleiohomeotic and GAGA factor for the maintenance of repression. Development.

[B12] Sangwan I, O'Brian MR (2002). Identification of a soybean protein that interacts with GAGA element dinucleotide repeat DNA. Plant Physiol.

[B13] Santi L, Wang Y, Stile MR, Berendzen K, Wanke D, Roig C, Pozzi C, Muller K, Muller J, Rohde W, Salamini F (2003). The GA octodinucleotide repeat binding factor BBR participates in the transcriptional regulation of the homeobox gene Bkn3. Plant J.

[B14] Meister RJ, Williams LA, Monfared MM, Gallagher TL, Kraft EA, Nelson CG, Gasser CS (2004). Definition and interactions of a positive regulatory element of the Arabidopsis INNER NO OUTER promoter. Plant J.

[B15] Kooiker M, Airoldi CA, Losa A, Manzotti PS, Finzi L, Kater MM, Colombo L (2005). BASIC PENTACYSTEINE1, a GA binding protein that induces conformational changes in the regulatory region of the homeotic Arabidopsis gene SEEDSTICK. Plant Cell.

[B16] Hulzink RJ, de Groot PF, Croes AF, Quaedvlieg W, Twell D, Wullems GJ, Van Herpen MM (2002). The 5'-untranslated region of the ntp303 gene strongly enhances translation during pollen tube growth, but not during pollen maturation. Plant Physiol.

[B17] Bao S, Corke H, Sun M (2002). Microsatellites in starch-synthesizing genes in relation to starch physicochemical properties in waxy rice (Oryza sativa L.). Theor Appl Genet.

[B18] Hardison RC (2000). Conserved noncoding sequences are reliable guides to regulatory elements. Trends Genet.

[B19] Guo H, Moose SP (2003). Conserved noncoding sequences among cultivated cereal genomes identify candidate regulatory sequence elements and patterns of promoter evolution. Plant Cell.

[B20] Inada DC, Bashir A, Lee C, Thomas BC, Ko C, Goff SA, Freeling M (2003). Conserved noncoding sequences in the grasses. Genome Res.

[B21] Hong RL, Hamaguchi L, Busch MA, Weigel D (2003). Regulatory elements of the floral homeotic gene AGAMOUS identified by phylogenetic footprinting and shadowing. Plant Cell.

[B22] Colinas J, Birnbaum K, Benfey PN (2002). Using cauliflower to find conserved non-coding regions in Arabidopsis. Plant Physiol.

[B23] Yang YW, Lai KN, Tai PY, Li WH (1999). Rates of nucleotide substitution in angiosperm mitochondrial DNA sequences and dates of divergence between Brassica and other angiosperm lineages. J Mol Evol.

[B24] Blanc G, Hokamp K, Wolfe KH (2003). A recent polyploidy superimposed on older large-scale duplications in the Arabidopsis genome. Genome Res.

[B25] Koch MA, Haubold B, Mitchell-Olds T (2000). Comparative evolutionary analysis of chalcone synthase and alcohol dehydrogenase loci in Arabidopsis, Arabis, and related genera (Brassicaceae). Mol Biol Evol.

[B26] Reyes JC, Muro-Pastor MI, Florencio FJ (2004). The GATA family of transcription factors in Arabidopsis and rice. Plant Physiol.

[B27] Soltis PS, Soltis DE, Chase MW (1999). Angiosperm phylogeny inferred from multiple genes as a tool for comparative biology. Nature.

[B28] The Gene Ontology Consortium (2000). Gene Ontology: tool for the unification of biology. Nat Genet.

[B29] Martin D, Brun C, Remy E, Mouren P, Thieffry D, Jacq B (2004). GOToolBox: functional investigation of gene datasets based on Gene Ontology. Genome Biol.

[B30] Lescot M, Dehais P, Thijs G, Marchal K, Moreau Y, Van de Peer Y, Rouze P, Rombauts S (2002). PlantCARE, a database of plant *cis*-acting regulatory elements and a portal to tools for in silico analysis of promoter sequences. Nucleic Acids Res.

[B31] PlantCARE database. http://bioinformatics.psb.ugent.be/webtools/plantcare/html/.

[B32] Higo K, Ugawa Y, Iwamoto M, Korenaga T (1999). Plant *cis*-acting regulatory DNA elements (PLACE) database: 1999. Nucleic Acids Res.

[B33] PLACE database. http://www.dna.affrc.go.jp/PLACE/signalscan.html.

[B34] Bolle C, Kusnetsov VV, Herrmann RG, Oelmuller R (1996). The spinach AtpC and AtpD genes contain elements for light-regulated, plastid-dependent and organ-specific expression in the vicinity of the transcription start sites. Plant J.

[B35] Arguello-Astorga GR, Herrera-Estrella LR (1996). Ancestral multipartite units in light-responsive plant promoters have structural features correlating with specific phototransduction pathways. Plant Physiol.

[B36] Pauli S, Rothnie HM, Chen G, He X, Hohn T (2004). The cauliflower mosaic virus 35S promoter extends into the transcribed region. J Virol.

[B37] Orozco BM, Ogren WL (1993). Localization of light-inducible and tissue-specific regions of the spinach ribulose bisphosphate carboxylase/oxygenase (rubisco) activase promoter in transgenic tobacco plants. Plant Mol Biol.

[B38] Goldsbrough AP, Albrecht H, Stratford R (1993). Salicylic acid-inducible binding of a tobacco nuclear protein to a 10 bp sequence which is highly conserved amongst stress-inducible genes. Plant J.

[B39] Pastuglia M, Roby D, Dumas C, Cock JM (1997). Rapid induction by wounding and bacterial infection of an S gene family receptor-like kinase gene in Brassica oleracea. Plant Cell.

[B40] Haberer G, Hindemitt T, Meyers BC, Mayer KF (2004). Transcriptional similarities, dissimilarities, and conservation of *cis*-elements in duplicated genes of Arabidopsis. Plant Physiol.

[B41] Teakle GR, Manfield IW, Graham JF, Gilmartin PM (2002). Arabidopsis thaliana GATA factors: organisation, expression and DNA-binding characteristics. Plant Mol Biol.

[B42] Schaffer R, Landgraf J, Accerbi M, Simon V, Larson M, Wisman E (2001). Microarray analysis of diurnal and circadian-regulated genes in Arabidopsis. Plant Cell.

[B43] Ma L, Sun N, Liu X, Jiao Y, Zhao H, Deng XW (2005). Organ-specific expression of Arabidopsis genome during development. Plant Physiol.

[B44] Ryals JA, Neuenschwander UH, Willits MG, Molina A, Steiner HY, Hunt MD (1996). Systemic Acquired Resistance. Plant Cell.

[B45] The *Arabidopsis *Genome Initiative (2000). Analysis of the genome sequence of the flowering plant Arabidopsis thaliana. Nature.

[B46] Arabidopsis genome in GenBank. ftp://ftp.ncbi.nih.gov/genomes/Arabidopsis_thaliana.

[B47] TAIR database. ftp://ftp.arabidopsis.org/home/tair/home/tair/Sequences/.

[B48] TIGR website. ftp://ftp.tigr.org/pub/data/b_oleracea/wgs_seq/.

[B49] Altschul SF, Madden TL, Schaffer AA, Zhang J, Zhang Z, Miller W, Lipman DJ (1997). Gapped BLAST and PSI-BLAST: a new generation of protein database search programs. Nucleic Acids Res.

[B50] The motified sputnik repeat-finder. http://capb.dbi.udel.edu/main/tools.htm.

[B51] Morgenstern B (1999). DIALIGN 2: improvement of the segment-to-segment approach to multiple sequence alignment. Bioinformatics.

[B52] Thompson JD, Higgins DG, Gibson TJ (1994). CLUSTAL W: improving the sensitivity of progressive multiple sequence alignment through sequence weighting, position-specific gap penalties and weight matrix choice. Nucleic Acids Res.

[B53] Koch M, Haubold B, Mitchell-Olds T (2001). Molecular systematics of the Brassicaceae: Evidence from coding plastidic matK and nuclear Chs sequences. Am J Bot.

[B54] Meyers BC, Lee DK, Vu TH, Tej SS, Edberg SB, Matvienko M, Tindell LD (2004). Arabidopsis MPSS: an online resource for quantitative expression analysis. Plant Physiol.

[B55] Arabidopsis MPSS database. http://mpss.udel.edu/at.

